# Demonstrative Midwifery

**Published:** 1850-02

**Authors:** 


### EDITORIAL DEPARTMENT.

*Demonstrative Midwifery.*—The subjoined correspondence, occasioned by the introduction of clinical, or demonstrative midwifery, in connection with the lectures on that branch of medicine in the Medical College of Buffalo, has been handed to us by the Chairman of the meeting, with a request that it be inserted in this Journal. We take pleasure in complying with this request. The illustration of labor with the living subject, is, doubtless, a novelty in this country. We are not aware that it has ever before been attempted. It enters, however, into the instruction of some foreign schools, constituting one of the features in which the latter are supposed to possess advantages over our domestic Institutions. Whatever may be the sentiments on the subject entertained by a portion of the community at large, (were it to be submitted to them,) the plan must, we think, commend itself to the cordial approbation of the medical profession; and, indeed, as it seems to us, the more intelligent members of any community, not excepting the female portion, must appreciate not alone the motives and the object, but its propriety in view of better preparing those soon to become practitioners of medicine, for the responsible duties of the Accoucheur. It should be stated that, during the demonstration, every regard was had to delicacy, the patient being entirely concealed from observation, except in so far as was requisite for the illustration. The privilege of being present was restricted to candidates for graduation, and medical gentlemen in attendance at the course of lectures; all of whom exhibited that degree of decorum so proper to the occasion.

The following is the correspondence referred to:—

University of Buffalo, Medical Department, Jan. 21, 1850.

The candidates for graduation having met pursuant to adjournment, W. B. Williams was appointed Chairman, C. C. Jewett, Secretary. The Report of the Committee was then called for. Whereupon, the Committee offered the following Preamble and Resolutions, which were adopted.

The Committee appointed at a meeting of the candidates of the class of 1849–50, for the purpose of expressing to Prof. White their sense of obligation for his recent and unusual efforts in our behalf, and to tender to him their thanks for extending to them advantages unprecedented in this country, would respectfully offer the following Resolutions:

*Resolved*, 1*st*. That in the recent successful endeavors of Prof. White to establish clinical teachings in connection with the instructions of his department, we have an invaluable addition to our already extended and liberal advantages from the Chair of Obstetrics.

2*d*. That we feel no ordinary degree of pride and congratulation, in claiming for the Medical Department of the University of Buffalo the honor of being the first, and, at present, the only among the American Schools of Medicine where Clinical instruction in Midwifery is rendered within the walls of the institution.

3*d*. That we tender to Prof. White our sincere thanks for his indefatigable efforts in rendering the subject of Obstetrics so simple and so plain, and especially in lately presenting for our instruction a case of *natural labor.*

C. C. VAN ANDEN,
JAS. S. HAWLEY,
JOHN ROOT,—*Committee.*

The Chairman and Secretary were instructed to present to Prof. White a copy of the proceedings of this meeting; and also to furnish a copy for publication in the Buffalo Medical Journal.

W. B. WILLIAMS, *Chairman.*

Charles C. Jewett, *Secretary.*

The following reply of Prof. White to the Committee has been handed to us with a request from the Committee that it be inserted in connection with the foregoing resolutions:—

University of Buffalo, Jan. 25, 1850.

Gentlemen:—

Your note containing a copy of the resolutions passed by the graduating class of the University of Buffalo, is just received.

Permit me to express my sense of obligation to yourselves and associates, for the very flattering notice you have been pleased to take of the recent successful effort to demonstrate to them a natural labor. Your approbation affords me sincere pleasure.

Though conceded by all to be a great desideratum, it was nevertheless an innovation, and likely to be opposed by popular prejudice, and without *your co-operation* it could not have been satisfactorily accomplished in the present instance, nor the hope of its repetition indulged.

Be assured therefore, that if any permanent progress has been made in the facilities for instruction in the important department, in which I have the honor to guide your investigations, it is mainly attributable to the serious decorum and the gentlemanlike deportment which was scrupulously observed by every member of the class on that occasion.

In the confident belief that with such an auspicious commencement, there will be little difficulty in furnishing the same much needed opportunity for observation to those who may succeed you;

I remain with sentiments of great regard, your friend and truly humble servant

JAMES P. WHITE.

To Messrs W. B. Williamr,
Charles C. Jewett, &c. &c.

